# The Role of Carbon in Metal–Organic Chemical Vapor Deposition-Grown MoS_2_ Films

**DOI:** 10.3390/ma16217030

**Published:** 2023-11-03

**Authors:** Tianyu Hou, Di Li, Yan Qu, Yufeng Hao, Yun Lai

**Affiliations:** 1 National Laboratory of Solid State Microstructures, School of Physics, College of Engineering and Applied Sciences, Jiangsu Key Laboratory of Artificial Functional Materials and Collaborative Innovation Center of Advanced Microstructures, Nanjing University, Nanjing 210023, China; 2 Key Laboratory of Photovoltaic and Energy Conservation Materials, Institute of Solid State Physics, Hefei Institutes of Physical Science, Chinese Academy of Sciences, Hefei 230031, China; 3 The Sixth Element (Changzhou) Materials Technology Co., Ltd. and Jiangsu Jiangnan Xiyuan Graphene Technology Co., Ltd., Changzhou 213161, China

**Keywords:** MOCVD, transition metal dichalcogenide, amorphous carbon, field-effect transistors

## Abstract

Acquiring homogeneous and reproducible wafer-scale transition metal dichalcogenide (TMDC) films is crucial for modern electronics. Metal–organic chemical vapor deposition (MOCVD) offers a promising approach for scalable production and large-area integration. However, during MOCVD synthesis, extraneous carbon incorporation due to organosulfur precursor pyrolysis is a persistent concern, and the role of unintentional carbon incorporation remains elusive. Here, we report the large-scale synthesis of molybdenum disulfide (MoS_2_) thin films, accompanied by the formation of amorphous carbon layers. Using Raman, photoluminescence (PL) spectroscopy, and transmission electron microscopy (TEM), we confirm how polycrystalline MoS_2_ combines with extraneous amorphous carbon layers. Furthermore, by fabricating field-effect transistors (FETs) using the carbon-incorporated MoS_2_ films, we find that traditional n-type MoS_2_ can transform into p-type semiconductors owing to the incorporation of carbon, a rare occurrence among TMDC materials. This unexpected behavior expands our understanding of TMDC properties and opens up new avenues for exploring novel device applications.

## 1. Introduction

Transition metal dichalcogenides (TMDCs) have attracted significant attention as promising materials for next-generation electronic and optoelectronic devices [1,2,3,4,5,6,7,8,9,10,11]. These applications heavily rely on the merits of TMDCs, including high carrier mobility [12,13], mechanical bandgap modulation [14,15,16], and spin valley coupling [17,18]. To meet the needs of modern electronics and optoelectronics, the scalable and atomically thin growth of TMDCs is a prominent challenge for the deposition of two-dimensional materials. To overcome the challenge, various synthesis methods have been explored, including chemical vapor deposition (CVD) [19,20,21,22], molecular beam epitaxy [23,24], and metal–organic chemical vapor deposition (MOCVD) [25,26,27,28,29,30]. In particular, MOCVD has shown potential in synthesizing wafer-scale TMDC films on insulating substrates, providing better control over film thickness and eliminating the need for transfer. During the MOCVD growth for large-scale TMDCs, particularly in situations where organic chalcogen precursors are desired as a less hazardous substitute for more toxic chalcogen hydrides, carbon will be inevitably introduced as an unintentional film impurity owing to pyrolysis side products from organic ligands [31]. However, the role of unintentional carbon incorporation is still a topic of ongoing debate. Some research teams reported that carbon can be incorporated through CH functionalization [32,33] or substitutional carbon doping [34], which are generally at the chalcogen sides of TMDC basal planes, or carbide transformation of TMDC edges [35]. Zhang et al. noted that “defective graphene” can be formed along with the formation of the TMDC layer, which hinders the lateral growth and the quality of TMDC films (e.g., continuity, stoichiometry, grain size, and phase purity) [31,36]. In this work, we investigate the growth of wafer-scale MoS_2_ films on sapphire substrates with metal–organic precursors of molybdenum hexacarbonyl (Mo(CO)_6_) and di-tert-butyl sulfide (DTBS, (CH_3_)_3_C)_2_S). Through analyses of Raman and PL spectra, we confirm the existence of co-deposited “amorphous carbon” during the growth of MoS_2_. In addition, atomic force microscopy (AFM) and transmission electron microscopy (TEM) reveal how polycrystalline MoS_2_ combines with extraneous amorphous carbon layers. Furthermore, by the fabrication of FETs, we find that even small amounts of unintentional carbon incorporated in the coalesced ultrathin MoS_2_ films, n-type MoS_2_, are transformed into p-type semiconductors, which is uncommon among TMDC materials. These findings show a new approach for the growth and integration of atomically thin TMDC films with process-induced carbon impurity doping, offering valuable insights into the promising prospects of utilizing this composite material for advanced electronic applications.

## 2. Materials and Methods

Sample preparation. MoS_2_ thin films were synthesized using a custom-made hot-wall reactor through the MOCVD technique. Sapphire substrates were positioned facing downwards on a quartz sample holder bar that was within a vertical quartz tube reactor chamber. The growth temperature was carefully controlled using a thermocouple and temperature controller. The reactor was then gradually heated at a rate of approximately 40 °C/min, while a 100 sccm flow of high-purity Ar (Air Liquide, Shanghai, China, Alphagaz 1, 99.999%) was maintained to create an inert environment. Once the desired growth temperature was reached, Ar was ceased, and the growth of MoS_2_ was initiated by vapor draw of molybdenum hexacarbonyl Mo(CO)_6_ (Sigma-Aldrich 577766, Beijing, China, >99.9% trace metal basis) and (CH3)_3_C)_2_S from separate containers, without the need for an additional carrier gas. Mo(CO)_6_ powder was placed on glass beads to increase surface area, and DES precursors were maintained at 30 and 12 °C, respectively. The flow rates of these precursors were precisely controlled using needle metering valves (SS-SS4-VH, Swagelok, Nanjing, China) and were determined based on their equilibrium vapor pressures. The nominal Mo(CO)_6_ flow rate was set at 0.02 sccm, and DES flow rates varied between 0.3 and 13.2 sccm. Furthermore, controlled amounts of high-purity H_2_ gas (Air Liquide, Alphagaz 1, 99.999%) with flows between 0 and 30 sccm were introduced into the system via a separate line, regulated by a mass flow controller. The entire process was conducted at working pressures ranging from 10^−2^ to 10^−1^ Torr. The growth temperature is 750 °C. Growth was stopped by cutting the Mo(CO)_6_, DES, and H_2_ flows off. The reactor was then cooled down to room temperature under 100 sccm Ar flow before the sample was removed. After each growth run, the reactor was annealed at 800 °C in Ar/H_2_ flow to eliminate the remaining reaction byproducts. Between runs, the reactor was maintained under a vacuum at a base pressure of around 1 × 10^−3^ Torr. During the loading and unloading of samples, an Ar flow of 150 sccm was employed to minimize exposure to ambient conditions.

Optical characterizations. An optical microscope (Nikon, Shanghai, China, Eclipse LV100ND) was utilized to take optical microscope images. A white balance was calibrated before taking the images. Raman and PL spectra were acquired using a WITEC optical microscopy (alpha 300R, Beijing, China) with a laser wavelength of 532 nm. The films were characterized under ambient conditions at 50× or 100× magnification with a spot size of approximately 1 μm. A low laser power of 0.1 mW was used to minimize heating effects and prevent optical doping and multiexciton dynamics in PL measurements. A 600 or 1200 grooves/mm grating was employed. Raman and PL measurements were conducted using integration times of 10 s and five accumulations. For each sample, five spots were measured along the sample diagonal in order to obtain the average data.

Structure characterizations. TEM (FEI Tecnai-G2 F20 operating at 200 kV, Beijing, China) was utilized to probe the atomic structure of the films. AFM was measured by NT-MDT NTEGRA Prima AFM, Shanghai, China. The chemical composition and stoichiometry of the films were investigated by XPS in an ultrahigh vacuum of 5 × 10^−10^ mbar using monochromatic Al Kα radiation with an energy of 1486.6 eV.

Film transfer. First, the as-grown films grown on sapphire substrates were coated with poly(methyl methacrylate) (PMMA, A4) by a spin coater, followed by a two-step process: 800 rpm for 15 s and 2000 rmp for 45 s. Then, the sample was baked at 180 °C for 2 min. Next, the samples were immersed in a diluted HF solution, and the PMMA/TMDCs assemblies were peeled off the substrate and floated on the HF solution. Then, the films were cleaned in deionized water for 20 min five times to guarantee the thorough elimination of the residual HF and transferred onto the desired substrate, such as the SiO_2_/Si substrate or patterned Au electrode. The sample was then put into a vacuum tank and dried at room temperature. Finally, the assembly was moved into the acetone and then isopropyl alcohol to dissolve the PMMA film.

## 3. Results and Discussion

**Figure 1:**
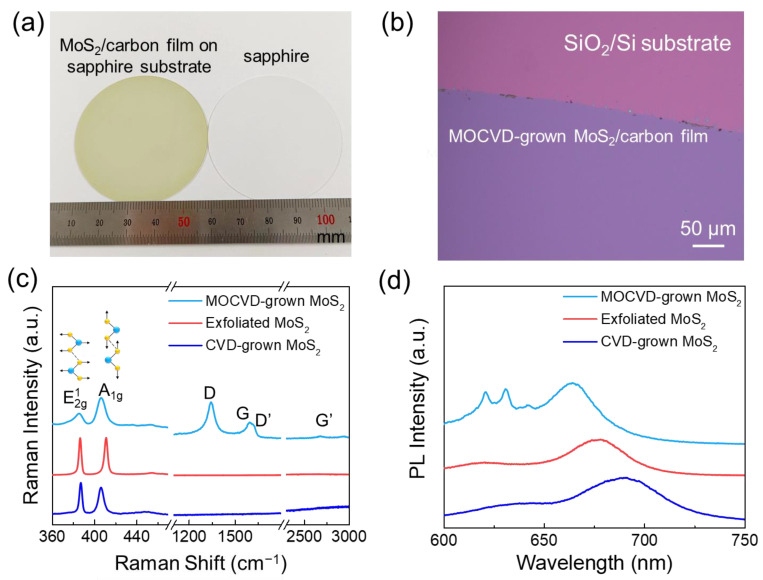
Optical characterizations of wafer-scale MOCVD-grown MoS_2_ films. (**a**) The photograph of MoS_2_ film grown on a sapphire substrate. (**b**) White-light microscope image of the as-grown large-area MoS_2_ film transferred onto the SiO_2_/Si substrate. (**c**) Raman spectra of transferred MOCVD-grown MoS_2_ films, exfoliated MoS_2_, and CVD-grown MoS_2_. (**d**) Photoluminescence spectra of the transferred MOCVD-grown MoS_2_ films, exfoliated MoS_2_, and CVD-grown MoS_2_.

Figure 1 displays a continuous and atomically thin film for wafer-scale MoS_2_ and exhibits intrinsic optical properties. As shown in Figure 1a, one can see a wafer-scale MoS_2_ film grown on a semi-polished 2-inch sapphire substrate. To enable measurements and applications of large-area uniform MoS_2_ and accurately determine the thickness of the MoS_2_ film, we use an etching-free, easy-processing, and large-area polymethyl methacrylate (PMMA)-assisted high-quality transfer strategy, which involves transferring the as-grown MoS_2_ film onto a SiO_2_/Si substrate, leveraging the hydrophilic behavior of the sapphire substrate and the accompanying capillary force [37]. In Figure 1b, we can observe the segments of MoS_2_ films after their wet transfer onto the SiO_2_/Si substrates. When examined under an enlarged optical microscope, the highly uniform color contrast showcases a homogenous thickness and in-plane continuity of the MoS_2_ film. The Raman spectroscopy shows a clear separation in the spectral domain, with peaks observed in the range of 360 cm^−1^ ≤ ω ≤ 420 cm^−1^ and 1300 cm^−1^ ≤ ω ≤ 3000 cm^−1^, as indicated in Figure 1c. Two characteristic peaks, $E_{2g}^{1}$ (~385 cm^−1^) and A_1g_ (~405 cm^−1^), confirm the presence of MoS_2_. These two phonon modes can be attributed to the in-plane displacement of both molybdenum and sulfur atoms ($E_{2g}^{1}$) and the out-of-plane displacement of the sulfur atoms (A_1g_), respectively [38]. The specific frequency difference of 20 cm^−1^ between $E_{2g}^{1}$ and A_1g_ peaks, commonly used as a layer thickness indicator, confirms the ultrathin nature of the obtained film compared to bulk MoS_2_ [39]. Additionally, the spectra in the range of 1300 cm^−1^ ≤ ω ≤ 3000 cm^−1^ suggest that the amorphous carbon co-deposits simultaneously with the MoS_2_ thin film, which originates from the pyrolysis of organic ligands of metal–organic precursors. The features of the G peak (~1595 cm^−1^) arising from the normal first-order Raman scattering process in graphene and the G’ band (~2680 cm^−1^) resulting from a second-order process indicate a graphene-like structure [40,41,42,43]. The high intensity of the disorder-induced D-band (~1345 cm^−1^) indicates that the carbon incorporated in the film is highly defective. The additional weak disorder-induced shoulder peak (D’ band) at ~1620 cm^−1^ and the D + D’ peak (~2940 cm^−1^) are also observed by Raman spectroscopy. Based on the D and G peak positions and an integrated I_D_/I_G_ intensity ratio (≈1.71), we assign this Raman feature to sp^2^ carbon, such as pyrolytic graphite [43,44], indicating that the MOCVD-grown sample is a MoS_2_/amorphous carbon composite film. In comparison, the characteristic peaks associated with amorphous carbon disappear in the exfoliated and CVD-grown MoS_2_. Figure 1d displays the PL spectroscopy, where two characteristic peaks of MoS_2_ (~660 nm and ~640 nm) are observed, which can be attributed to the A and B direct bandgap optical transitions [45]. However, in addition to these intrinsic characteristic peaks of MoS_2_, two other distinctive and non-negligible peaks at ~620 nm and ~630 nm are observed in comparison with the exfoliated MoS_2_ and CVD-grown MoS_2_, which may be induced by the Raman shift of the G’ and D + D’ bands in the amorphous carbon.

**Figure 2:**
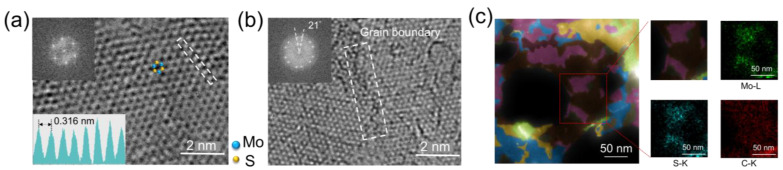
Structure characterizations of the transferred MoS_2_/carbon composite film. (**a**) The high-resolution bright-field TEM image of a freely suspended MoS_2_/carbon composite film on a TEM grid. The inset shows the FFT pattern of the TEM image and the intensity profile of the corresponding white dashed box. (**b**) The grain boundary is highlighted by the white dashed box with a rotation angle of ~21°, as confirmed by the FFT pattern shown in the inset. (**c**) The false color-coded HAADF-TEM image. Corresponding EDS elemental maps display the composition and distribution of each chemical element.

In order to examine the atomic structure and morphology of the MoS_2_/amorphous carbon composite film, high-resolution TEM imaging and AFM provide a distinctive atomic-level perspective of the MoS_2_ and carbon combination (shown in Figure 2). As depicted in Figure 2a, the atomic structure of the MoS_2_/carbon composite film is characterized by aberration-corrected high-resolution TEM. The image reveals a well-organized honeycomb lattice with an interatomic distance of approximately 0.316 nm, which is consistent with previous observations for MoS_2_ [46,47]. The lattice consists of hexagonal rings formed by alternating molybdenum and sulfur atoms. Owing to the contrast of bright-field TEM image scales that are roughly at the square of atomic number Z [48], the brighter atomic spots are molybdenum sites, and the dimmer ones are the two stacked sulfur atoms, as indicated by the top-view schematic. The fast Fourier transform (FFT) pattern in the inset of Figure 2a reveals only one set of six-fold symmetry diffraction spots, suggesting a hexagonal arrangement. And the lattice spacing of 0.27 nm can be assigned to (1 0 0) planes. In Figure 2b, a grain boundary is highlighted within the white dashed box. The FFT pattern reveals two sets of six-fold symmetrical diffraction spots with a rotation angle of ~21°, indicating the presence of two grains in this region. Figure 2c demonstrates the high-angle annular dark field transmission electron microscopy (HAADF) image of the crystal structure for the corresponding composite. The composite image is obtained from overlapping false color-coded HAADF-TEM images, where the color contrast corresponds to different domains. The intersections of the grains constitute many faceted tilt and twisted boundaries, arising from disordered crystals that form randomly oriented polycrystalline aggregates. As shown in the legend, the black areas are holes of the TEM grid, the brown and grey areas correspond to carbon films, and the other colorful areas represent MoS_2_ on the carbon films. Energy-dispersive X-ray spectroscopy (EDS) elemental maps also show the chemical composition and are zoomed-in in the HAADF image. The maps reveal that the carbon element uniformly distributes throughout the detection area, while Mo and S are vertically localized within their corresponding irregular domains. This indicates that carbon layers form on top of MoS_2_ without forming any inter- or in-plane chemical bonds. Therefore, the interaction between these layers is primarily governed by van der Waals forces.

**Figure 3:**
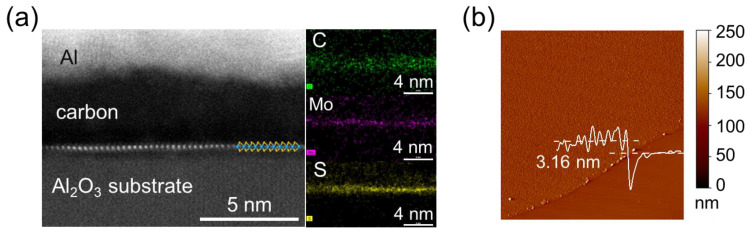
(**a**) The cross-sectional TEM of the MoS_2/_amorphous carbon composite film grown on sapphire. The EDS elemental maps are on the right panel. (**b**) The AFM image of the MoS_2_/amorphous carbon composite film edge transferred onto the SiO_2_/Si substrate.

To further confirm the growth process of the MoS_2_/carbon film, the cross-sectional TEM samples are fabricated by focused ion beam (FIB) milling. A thin layer of aluminum is thermally evaporated on top of the sample to enhance the color contrast with carbon. A thin layer of gold is thermally evaporated to prevent the oxidation of aluminum and enhance conductivity. The cross-sectional TEM of the sample (Figure 3a) confirms the presence of MoS_2_ with pristine interfaces and reveals the carbon layers above the MoS_2_. The thickness of the MoS_2_/carbon film is approximately 3 nm, which is consistent with the corresponding height profile value of ~3.16 nm shown in the AFM image of the sample edge (Figure 3b).

**Figure 4:**
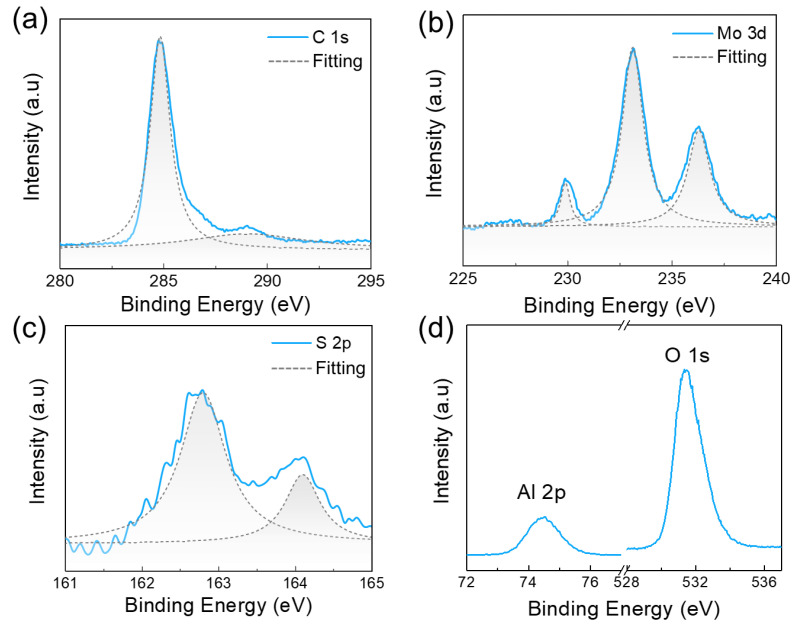
XPS spectra of the MOCVD-grown MoS_2_/carbon film on the sapphire substrate. The XPS spectrum of (**a**) the C 1s orbit, (**b**) the Mo 3d orbit, and (**c**) the S 2p orbit of MOCVD-grown MoS_2_/carbon films. (**d**) The XPS spectrum of the sapphire substrate.

To assess the elemental composition of our MOCVD-grown samples, we perform X-ray photoelectron spectroscopy (XPS) measurements of the films. The XPS spectra of films grown on sapphire are displayed in Figure 3. As shown in the legend, in addition to the prominent peaks corresponding to Mo, S, and C elements (Figure 4a–c), signals related to O and Al are also observed (Figure 4d). This suggests good surface coverage of the substrate and the formation of large-area continuous MoS_2_ layers. The XPS spectrum of the Mo 3d core level (Figure 4b), which corresponds to the expected energy positions of the MoS_2_ film, exhibits correct splitting spin orbitals, displaying two main peaks at approximately 229.6 eV (Mo 3d_5/2_) and 232.8 eV (Mo 3d_3/2_) for the 2H phase, respectively. These peaks are characteristic of Mo^4+^ in MoS_2_ [49,50,51,52,53,54,55,56]. Additionally, the MoS_2_ film on sapphire exhibits a minor but discernible peak at ~227.1 eV, which is attributed to the presence of S 2s or the molybdenum carbide (Mo-C bond) [50,56]. Another notable peak at a higher binding energy in the Mo 3d core level spectrum is related to the Mo^6+^, which arises from the oxidation of MoO_3_ [49]. Regarding the S 2p region (Figure 4c), the binding energy peaks observed at 162.5 eV and 163.8 eV correspond to the S 2p_3/2_ and 2p_1/2_ core orbitals, respectively, further confirming the presence of MoS_2_ in the 2H phase [50]. The XPS survey spectrum of the C 1s orbital exhibits two main peaks centered at 284.6 eV and 288.8 eV [49,55], where the major peak at 284.6 eV represents the hybridization of sp^2^ bond (Figure 4a). It indicates the presence of graphene and further verifies that our MOCVD-grown sample is a MoS_2_/carbon composite film. However, the minor but unique peak (288.8 eV) remains dim; therefore, we cannot definitively conclude that the peak at ~227.1 eV in the Mo 3d binding energy represents the Mo-C bond.

**Figure 5:**
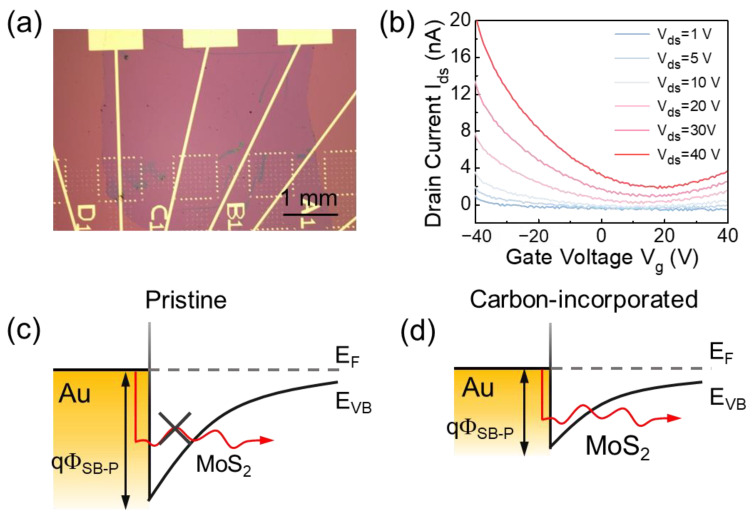
Electrical characterization of MoS_2_/carbon FETs. (**a**) The optical micrograph of the MoS_2_/carbon film transferred onto the patterned SiO_2_/Si substrate. (**b**) Typical transfer characteristics (I_d_-V_g_) of as-fabricated MoS_2_/carbon FETs. Schematics of the valence band position (E_VB_) near the source for pristine (**c**) and carbon-incorporated (**d**) FET devices. For the pristine devices, the hole conduction is prevented by a wide Φ_SB-P,_ so the device shows n-type behavior. However, after carbon incorporation, the Au Fermi level pins closer to the VB of MoS_2_, leading to a smaller Φ_SB-P_ for the hole injection.

To investigate the electrical properties of the MoS_2_/carbon composite films, we transfer the films onto a pre-patterned gold electrode (Figure 5a). It can be found that the transfer curves show a notable decrease in behavior in the left section when the gate voltage varies from −40 to 40 V (Figure 5b), indicating an “off” process. The right section shows a gradual increase in current, which can be interpreted as an “on” process. Although the current does not drop to the −12 or even −13 power of a completely off state, the source drain current exhibits a switching behavior, with a maximum current of up to 20 nA. It is important to note that the carbon incorporation results in the conversion of the MoS_2_ film from a typical n-type semiconductor to a p-type semiconductor. The pristine MoS_2_ electric devices generally display unipolar n-type behavior. However, the carbon-incorporated devices exhibit a p-type current compared to pure MoS_2_ devices. This can be attributed to the influence of carbon within the composite films, altering the semiconducting properties of MoS_2_. As shown in Figure 5c, for pristine TMDC devices, the metal Fermi level aligns more closely with the conduction band (CB) than the valence band (VB), leading to a diminished electron Schottky barrier (Φ_SB-n_) compared to the hole Schottky barrier (Φ_SB-P_) [57]. After carbon incorporation, the incorporation will shift from the Fermi level to the VB. This realignment of the metal MoS_2_ Fermi level pinning leads to a reduction in Φ_SB-P_ (Figure 5d). [32,58]. As theoretically calculated by A. Chanana et al. [59], the Φ_SB-P_ for the MoS_2_-Au interface is 1.2 eV. When inserted by graphene, the Φ_SB-P_ for MoS_2_–graphene–Au interface decreases to 1.14 eV; namely, the Fermi level shifts to VB. The smaller Schottky tunnel barrier no longer impedes hole injections [32]. Therefore, carbon impurities alter the electronic or optoelectronic properties of our composite TMDC films. The carrier concentration can be estimated by the following formula [60]:

*n* = C_bg_(V_bg_ − V_th_)/e,

C_bg_ = ε_0_ε_r_/d,

where C_bg_ ≈ 1.2 × 10^−4^ F/m^2^ is the gate capacitance per unit area for 285 nm SiO_2_ dielectric, V_bg_ is the back gate voltage, V_th_ is the threshold voltage for the channel to start accumulating charge and conducting, e is the elementary charge, ε_0_ is the permittivity of free space, ε_r_ is the relative permittivity, and d is the thickness of the dielectric layer. A back gate voltage can be applied to the conducting Si substrate to modulate the MoS_2_ carrier concentration. When V_bg_ is 40 V, the carrier concentration is calculated to be 1.2 × 10^11^ cm^−2^.

## 4. Conclusions

In conclusion, we comprehensively investigate the structure, optical, and electrical properties of a large-scale and coalesced MoS_2_/carbon composite film. Compared to CVD-grown and exfoliated MoS_2_, MOCVD-grown MoS_2_ can introduce unintentional carbon incorporation, leading to the shift of the PL characteristic peak, the emergence of carbon features in Raman properties, the alteration of the (opto)electronic properties, etc. Specifically, through Raman and PL spectra, we verify the presence of carbon incorporation. Additionally, cross-sectional TEM and XPS are employed to observe the combination of carbon and the MoS_2_ material. Furthermore, by fabricating FET devices, we explore the influence of carbon on the electric performance of the composite film. We find that the general n-type MoS_2_ can be converted to a p-type semiconductor owing to the incorporation of carbon in the MoS_2_ film. A comparison chart is tabulated in Table 1. Our study provides a valuable understanding of process-induced C impurity doping in MOCVD-grown two-dimensional semiconductors and might have important influences on advanced electronic applications.

## Figures and Tables

**Table 1:** Comparison chart of the properties with other methods used for MoS_2_.

	Carbon Incorporated or Not	Precursors	Raman [61,62]	PL [61,63]	Semiconducting Property
MOCVD-grown MoS_2_	yes	Mo(CO)_6_, (CH_3_)_3_C)_2_S	$E_{2g}^{1}$(~385 cm^−1^), A_1g_ (~405 cm^−1^) and some carbon related peaks	A (~660 nm), B (~640 nm)	p-type
CVD-grown MoS_2_	no	MoO_3_ and S powders	$E_{2g}^{1}$(~385 cm^−1^), A_1g_ (~403 cm^−1^)	A (~660 nm)	n-type
exfoliated MoS_2_	no	bulk materials	$E_{2g}^{1}$(~385 cm^−1^), A_1g_ (~403 cm^−1^)	A (~660 nm), B (~610 nm)	n-type

## Data Availability

Not applicable.
